# Asymptomatic and yet *C. difficile*-toxin positive? Prevalence and risk factors of carriers of toxigenic *Clostridium difficile* among geriatric in-patients

**DOI:** 10.1186/s12877-016-0358-3

**Published:** 2016-11-15

**Authors:** Klaus Nissle, Daniel Kopf, Alexander Rösler

**Affiliations:** 1 Medical Centre (MVZ) of the Katholisches Marienkrankenhaus gGmbH/Laboratory Medicine (ILMT), Alfredstraße 9, 22087 Hamburg, Germany; 2 Katholisches Marienkrankenhaus gGmbH/Geriatric Clinic, Alfredstraße 9, 22087 Hamburg, Germany

**Keywords:** *Clostridium difficile* colonization, Asymptomatic carrier, Prevalence, Risk factors, Geriatrics, Geriatric assessment

## Abstract

**Background:**

*Clostridium difficile* infections (CDI) are the most frequent cause of diarrhoea in hospitals. Geriatric patients are more often affected by the condition, by a relapse and complications. Therefore, a crucial question is how often colonization with toxigenic *Clostridium difficile* strains occurs in elderly patients without diarrhoea and whether there is a “risk pattern” of colonized patients that can be defined by geriatric assessment. Furthermore, the probability for those asymptomatic carriers to develop a symptomatic infection over time has not been sufficiently explored.

**Methods:**

We performed a cohort study design to assess the association of clinical variables with *Clostridium difficile* colonization. The first stool sample of 262 consecutive asymptomatic patients admitted to a geriatric unit was tested for toxigenic *Clostridium difficile* using PCR (GeneXpert, Cepheid). A comprehensive geriatric assessment (CGA) including Barthel Index, Mini Mental State Examination (MMSE) and hand grip-strength was performed. In addition, Charlson Comorbidity Index, body mass index, number and length of previous hospital stays, previous treatment with antibiotics, institutionalization, primary diagnoses and medication were recorded and evaluated as possible risk factors of colonization by means of binary logistic regression. Secondly, we explored the association of *C. difficile* colonization with subsequent development of CDI during hospital stay.

**Results:**

At admission, 43 (16.4%) patients tested positive for toxin B by PCR. Seven (16.3%) of these colonized patients developed clinical CDI during hospital stay, compared to one out of 219 patients with negative or invalid PCR testing (Odds ratio 12,3; Fisher’s exact test: *p* = 0.000). Overall, 7 out of 8 (87.5%) CDI patients had been colonized at admission. Risk factors of colonization with *C. difficile* were a history of CDI, previous antibiotic treatment and hospital stays. The parameters of the CGA were not significantly associated with colonization.

**Conclusion:**

Colonization with toxigenic *Clostridium difficile* strains occurs frequently in asymptomatic patients admitted to a geriatric unit. Previous CDI, antibiotic exposure and hospital stay, but not clinical variables such as CGA, are the main factors associated with asymptomatic *Clostridium difficile* carriage. Colonization is a crucial risk factor for subsequent development of symptomatic CDI.

## Background

*Clostridium difficile* infections (CDI) have become the most frequent cause of diarrhoea in hospitals and care facilities [1]. Higher age, recent hospitalization, previous treatment with antibiotics, previous CDI, immunosuppression, proton pump inhibitor (PPI) use, surgical interventions, living in a care facility and known comorbidities are all associated with the development of a CDI [2–6]. Relapses and multiple recurrences constitute an increasing problem [7–10].

CDI patients have a 2.5 times increased 30-day mortality compared to in-patients without diarrhoea; the CDI-related mortality is approximately 10% [11]. In geriatric patients, the severity of the disease course, the recurrence rates and the mortality are even higher [12–14].

The pathogen causing the symptomatic CDI may be present at admission, or it may be acquired during the hospital stay. Colonization rates for geriatric departments have not yet been investigated.

Neither has it been conclusively established in which way asymptomatic carriage influences the risk of a symptomatic CDI disease nor to what degree it plays a relevant role in the spreading of the pathogen [15]. Since geriatric patients are often affected by CDI with a tendency to serious progression and recurrence [16–19], we examined patients at the moment of admission to a geriatric ward for the prevalence of asymptomatic toxigenic *C. difficile* carriage including the causal risk factors. We explored whether a risk pattern for carriage can be defined within the geriatric assessment and how likely it is for asymptomatic carriers to develop a symptomatic CDI during their hospital stay.

## Methods

The study was designed as a cohort study. Following approval (PV4643) by the ethics committee of the Ärztekammer Hamburg (Hamburg’s General Medical Council), 262 patients without diarrhoea consecutively admitted to the geriatric unit of the Marienkrankenhaus from March to November 2014 were examined. The Katholisches Marienkrankenhaus gGmbH is a teaching hospital of the University of Hamburg with 550 inpatient beds in total in various different medical units. The geriatric department consists of 5 wards of 126 beds. Written consent was obtained after detailed information and explanation of the study procedures.

First, we tried to assess the association of clinical variables with *Clostridium difficile* colonization.

Secondly, we explored the association of *C. difficile* colonization with subsequent development of CDI. Patients were monitored throughout their hospital stay with regard to the development of a symptomatic CDI.

As described in 2.5, Statistical analyses, a required number of at least 250 patients had been calculated based on an examined rate of CDI of 4% in geriatric in-patients in 2012 in the Katholisches Marienkrankenhaus.

Only patients without diarrhoea were included with diarrhoea being defined as the occurrence of >3 unformed stools per day. Participation could be revoked at any time without stating any reasons. Stool samples at the first bowel movement after hospital admission were collected for testing. They were to be delivered within 6 days after admission obtained spontaneously; later stool samples were not evaluated.

Patients who had acute diarrhoea, or who declined participation, or who were incapable of giving consent and had no legal representative, or whose legal representative refused consent were excluded.

Patients who agreed to take part but failed to deliver a stool sample within the first 6 days after admission were excluded as well. The first stool sample of each participant was tested for toxigenic *Clostridium difficile* using PCR (see Lab analysis.).

### Data collection

The following anamnestic data of the patients taking part were recorded: age, sex, date of admission, current duration of hospital stay, number and duration of previous hospital stays within the past 6 months, current or previous treatments with antibiotics within the past 6 months and the respective agents, medication with proton pump inhibitors (PPI) or immunosuppressants. In our patients the following immunosuppressant drugs were used: systemic corticosteroids, methotrexate, ciclosporine and leflunomide; two patients had just finished a cycle of cytostatic medication with R-CHOP (including rituximab, cyclophosphamide, vincristine, doxorubicine and prednisolone).

The immediately previous place of residence was categorized as transfer from 1) a stay at an external hospital, 2) a different department of the same hospital, 3) home, which also included a care facility. Further, the place of residence before admission to hospital was recorded: living independently, in sheltered accommodation, or within a care facility; in addition, known previous CDI episodes (medical history, referral letters) and one further category, “post-surgery” (surgery as the initial reason for admission to hospital), were included, assuming a higher risk of colonization in this patient group.

### Geriatric assessment at admission

At the time of admission, all participating patients underwent the following geriatric assessment: Barthel Index [20], Mini Mental State Examination (MMSE) [21], measuring Handgrip Strength in kPa [22], and Timed up and Go [23]. Additionally, the Body Mass Index was determined and the presence of comorbidities was recorded using the Charlson Comorbidity Index [24].

### Lab analysis

In all participating patients, the first stool sample after admission to the geriatric ward was tested for toxigenic *C. difficile* using PCR. A commercially available PCR test system was used: the Xpert *C. difficile* test on the GeneXpert by Cepheid (Germany, Frankfurt).

This test uses real-time PCR to determine the presence of toxigenic *C.difficile* strains by means of nucleic acid detection of gene sequences of the cytotoxin B (tcdB) and of the binary toxin (cdt), and also the deletion of the repressor gene tcdC. This deletion leads to an increased toxin production and is characteristic of the PCR-ribotype 027 (PFGE-type NAP1 and REA-type B1).

The result either confirms or rules out the existence of toxigenic *C.difficile*; if a positive result occurs, additional information is given on whether the pathogenic strain ribotype 027 (PFGE-type NAP1, REA-type B1) is likely to be present or not.

The performance data provided by the test manufacturer compared with the toxigenic culture result in a sensitivity of 100% at a specificity of 93% (Xpert *C. difficile*; GXCDIFFICILE-CE-10; 300-9291G Rev E, November 2012).

In patients who subsequently developed clinical symptoms of CDI, an EIA test for glutamate dehydrogenase (GDH) and, if positive, a follow-up toxin A/B EIA test were performed (C.DIFF CHEK–60 and *C. DIFFICILE* TOX A/B II; TECHLAB/ Alere USA).

### Surveillance of the occurrence of a symptomatic CDI

For clinical follow-up, *Clostridium difficile* infection (CDI) was defined as diarrhoea with loose stools at least three times in 24 h, elevated C-reactive protein and blood leukocyte count, and positive results of GDH and toxin A/B EIA tests as described above.

Occurring diarrhoea was documented throughout the entire hospital stay. When diarrhoea occurred, the diagnostic lab tests mentioned above were carried out to either confirm or rule out a CDI.

At discharge the patient file was evaluated and the marked fact of a CDI during the stay in the geriatric ward was recorded for the study.

### Statistical analysis

The sample size calculation was based on the follow-up part of the study: Preliminary examinations in 2012 showed that 4% of all in-patients of the geriatric department in our hospital suffered from clinical CDI. As this rate is in accordance with a literature report in a comparable institution [25], we based our sample size calculation on this assumption. In addition, we assumed that 20% of our patients would be colonized carriers at admission, but asymptomatic [2, 26]. We aimed to detect a tenfold increase of clinical infection rate in carriers vs. non-carriers by means of univariate analysis with a power of 80% at a level of significance (α) of 5%. Based on these assumptions, sample size calculation yielded a sample size of 250.

First, a descriptive characterization of the participants was made. As a next step, clinical variables were compared between the PCR positive and PCR negative groups by univariate analysis. Statistical significance was reached for a bilateral *p* value ≤ 0.05. *Clostridium difficile* colonization (PCR positive) was treated as dependent variable while clinical features, such as previous antibiotic treatment and CGA domains, were treated as independent variables.

In addition, the significant variables of this univariate analysis were entered into the binary logistic regression (“backwards: LR” method) in order to predict the PCR results. As level of significance for variable entry *p* ≤ 0.05 was used. The level used for the backward method was p(out) = 0.10, p(in) = 0.05.

The correlation matrix of the significant variables was calculated and used for variable entry into the model in order to minimize the risk of overfitting and multicollinearity.

Thus, only significant variables of the univariate analysis were chosen for the multivariate analysis. The calculation was carried out by an independent statistician using SPSS, version 22.0, according to Schendera [27].

Finally, we compared the initially colonized patients who developed CDI with the initially colonized patients who did not fall ill during hospital stay by cross tabulation and Fisher’s exact test (two- sided, *p* = 0.000).

## Results

**Fig. 1:**
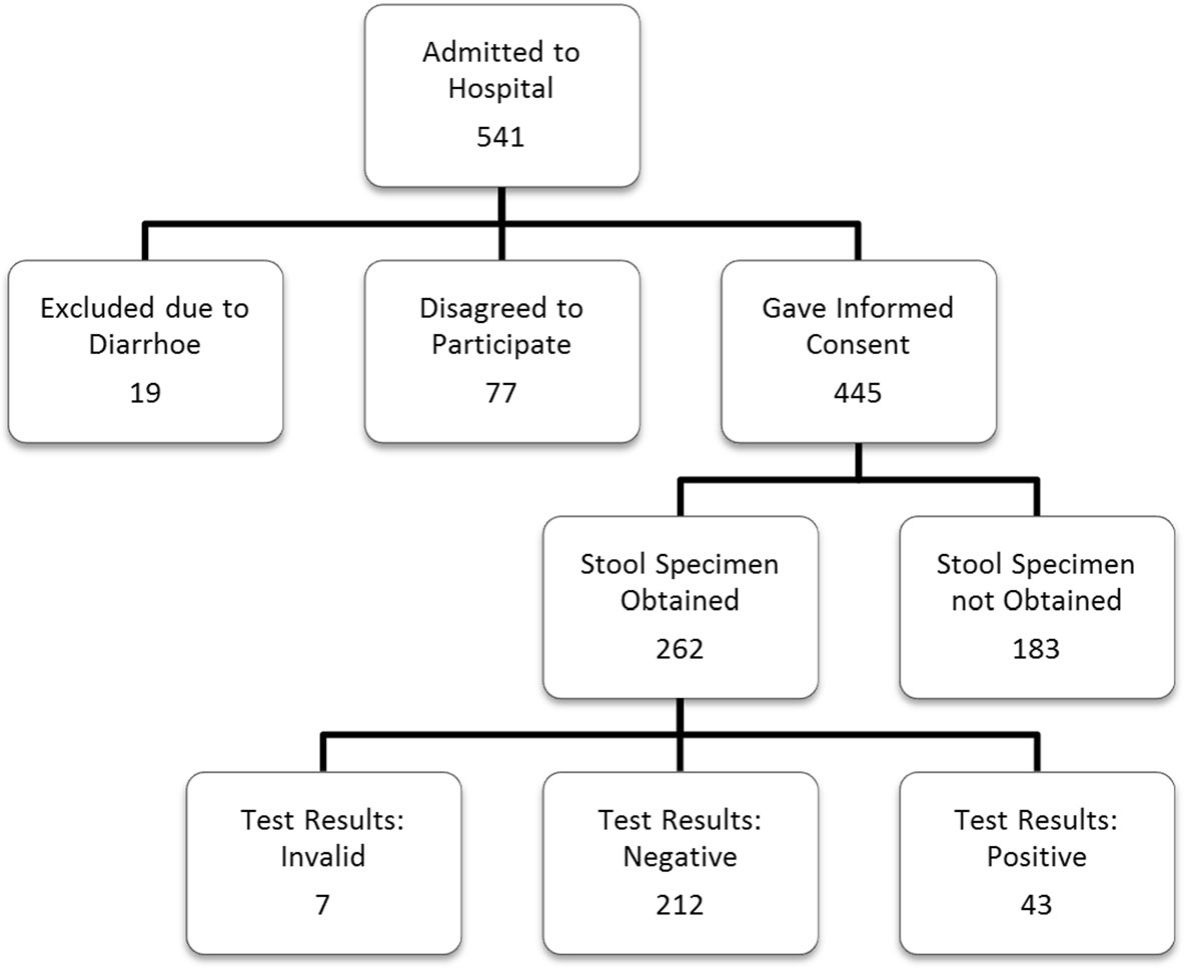
Recruitment and Participation

During the investigation period of March to November 2014, 541 patients admitted to the geriatric department of the Marienkrankenhaus Hamburg were invited to take part in the *C.difficile* study. Of these 541 possible participants, 77 refused and 19 patients were excluded due to acute diarrhoea. Therefore, 445 patients were potential participants in the study. Of this group, 183 patients failed to send in a stool sample within 6 days of admission. Most of these patients were mobile and forgot to take a sample, some of them suffered from constipation. Both groups showed a similar distribution in ADL (Barthel Index) and cognition (Mini Mental State Examination). The number of patients participating and examined using PCR thus amounted to 262 (Fig. 1; 165 women, 97 men).

Most patients (48.5%) had been transferred from other departments of the Marienkrankenhaus (mainly internal medicine, orthopaedic surgery or neurology), 29.8% from various departments of other hospitals. The remaining 21.8% of the patients had been admitted from their homes either as elective or as emergency cases.

In Germany, about 80% of admissions to a geriatric ward happen post-acute to restore pre-admission functional status. About 20% of patients are admitted direct, for example from the emergency department or the general practitioner. However, the common characteristics of geriatric patients are multimorbidity and age, usually over 65 years. The usual length of stay is about 2 to 3 weeks.

The characteristics of patients included in the study are shown in Table 1.

**Table 1:** Demographics, epidemiological data and clinical characteristics of patients included in the study

Characteristic	Minimum	Maximum	Mean ± Standard deviation	Percentage
Age	59	100	78.8 ± 7.7	
Duration of previous hospital stays within past 6 months [days]	0	180	25.3 ± 27.1	
Number of previous hospital stays within past 6 months	0	5	1.5 ± 1.1	
Duration of current stay in geriatric department [days]	1	48	16.4 ± 6.7	
Time of stool sample after admission [days]	0	6	2.5 ± 1.6	
Mini Mental State Examin. /30	0	30	25.1 ± 5.1	
Barthel Index/100	0	100	50.9 ± 24.1	
Timed Up & Go [Sec]	6	58	23.7 ± 10.9	
Charlson CM Index	0	12	3.3 ± 2.4	
Handgrip Strength [kPa]	8	86	40.5 ± 16.2	
BMI	14.1	51.8	25.8 ± 8.0	
PPI premedication				64.5%
Antibiotics within past 6 months				66%
Immunosuppression				16%
Previous episodes of CDI				2.7%
“Post-Surgery” hospital stay				26.7%
Place of residence care facility				8.8%

There was no difference in gender distribution among participants and non-participants; on average, participants were 1.5 years younger than non-participants.

Of all 262 stool samples tested using PCR, 212 (80.9%) showed a negative result, 43 (16.4%) showed a positive result, and 7 (2.7%) tests yielded an invalid result despite repeated analysis. The stool samples tested “invalid” did not differ from the valid tested samples in other respects.

Statistical evaluation of C*.difficile* carriage is shown in Table 2.

**Table 2:** Univariate and multivariate analyses of toxigenic *C. difficile* carriage identified by PCR

Characteristic	PCR positive Mean ± standard deviation	PCR negative Mean ± standard deviation	*P* value (univariate analysis) (*significant)	Odds ratio (multivariate analysis) (95% confidence interval)
Age	80.49 ± 8.93	78.54 ± 7.20	0.123	
Day of stool sample (day after admission)	2.58 ± 1.68	2.42 ± 1.64	0.568	
Length of stay in days	17.14 ± 8.25	16.29 ± 6.41	0.454	
Number hospital stays past 6 months	2.07 ± 1.22	1.32 ± 1.00	<0.001*	1.6 times per hospital stay (1.1–2.2)
Length hospital stays past 6 months	39.88 ± 26.43	22.47 ± 26.39	<0.001*	^a^
Mini Mental State Examination	25.19 ± 4.99	25.10 ± 5.06	0.920	
Barthel Index	47.38 ± 23.48	50.86 ± 23.95	0.390	
Timed Up & Go	27.81 ± 11.57	23,38 ± 10.83	0.084	
Charlson CM Index	3.91 ± 2.71	3.12 ± 2.25	0.044*	^b^
Handgrip Strength	36.95 ± 13.68	41.24 ± 16.62	0.123	
BMI	24.65 ± 6.20	26.22 ± 7.98	0.224	
Characteristic	PCR positive Women/Men	PCR negative Women/Men	*P* value (univariate analysis) (*significant)	Odds ratio (multivariate analysis) (95% confidence interval)
Sex	65.1%/34.9%	62.3%/37.7%	0.726	
Characteristic	PCR positive Yes/No	PCR negative Yes/No	*P* value (univariate analysis) (*significant)	Odds ratio (multivariate analysis) (95% confidence interval)
Immunosuppression	14.0%/86.0%	15.6%/84.4%	0.790	
Residence care facility	18.6%/81.4%	6.6%/93.4%	0.010*	^b^
Previous treatment with antibiotics past 6 months	93.0%/7.0%	60.8%/39.2%	<0.001*	3.7 times (1.0–13.9)
Previous episodes of CDI	11.6%/88.4%	0.9%/99.1%	<0.001*	12.3 times (1.9–81.6)
PPI premedication	76.7%/23.3%	62.3%/37.7%	0.071	
“Post–Sugery” hospital stay	53.5%/46.5%	21.2%/78.8%	<0.001*	3.5 times (1.4–8.8)

^a^length of hospital stay correlated by *R* = 0.58 to the variable “number of hospital stays” and was therefore excluded from the final multivariate model

^b^not statistically significant in the multivariate model

The univariate analyses showed significant differences between PCR positive and PCR negative results (*p* ≤ 0.05) with the following variables:

Number and length of hospital stays within the past 6 months, transfer from hospital stay, treatment with antibiotics within the past 6 months, occurrence of previous episodes of CDI, transfer from care facility, primary diagnosis postoperative stay, Charlson Comorbidity Index (Table 2). Therefore *C. difficile* colonization expressed as a positive PCR result was treated as dependent variable and clinical features (characteristics) listed in Table 2 as independent variables. Length of hospital stay correlated by *R* = 0.58 to the variable hospital stays and was therefore excluded from the final multivariate model.

The Charlson Comorbidity Index was significantly associated with a positive CD-PCR (*p* = 0.044). However, the increase of probability was small (OR 1.14; 95% CI: 1.002–1.307).

Further evaluated characteristics, especially further parameters of the geriatric assessment, did not correlate significantly (*p* > 0.1) with the PCR result.

Significant characteristics for the prediction of positive PCR result in the multivariate model were the number of previous hospital stays, a previous episode of CDI, taking antibiotics within the past 6 months and the diagnosis “post-surgery hospital stay”.

Only in 1 of 43 positive test results did the test system indicate the presence of pathogenic ribotype 027.

During their hospital stays, 8 out of 262 participants developed a symptomatic CD infection. Seven of these 8 symptomatic patients had been found colonized at admission (87.5%). Of the 43 patients who tested positive at admission, 7 developed a symptomatic CDI (16.3%).

In the 7 initially colonized and over time symptomatic CDI patients, no significant differences were found with regard to the initially recorded risk parameters, compared to the 36 patients who were colonized and did not fall ill in the course of the study. This may be caused by the small number of CDI cases, so none of the initially analysed clinical variables reached statistical significance. The patients were not monitored during their hospital stay with regard to different clinical characteristics.

## Discussion

In this study, approximately one in every six patients admitted to the geriatric unit without diarrhoea (16.4%) arrived colonized with toxigenic *Clostridium difficile* strains. So far, no comparable data have been shown for patients in geriatric care. The number of asymptomatic CD-colonized patients ranges from 0.6% in the hospital [28] up to 51% in care facilities [29]. Rates of colonization of in-patients were reported as being between 9.7 and 15% [2, 26, 30–32]. Therefore, colonization rate of our geriatric patients, 9% of whom were admitted from nursery homes and were impaired in their activities of daily living (average Barthel Index 50/100), lies within the expected range.

We identified risk factors for asymptomatic carriage. Some of them were already known as risk factors for the development of a symptomatic CDI [33]. Of these, previously experienced CDI episodes had the highest impact (OR 12.3; 95% CI: 1.9–81.6), followed by previous treatments with antibiotics (OR 3.7; 95% CI: 1.0–13.9) and the primary diagnosis “post-surgery” (OR 3.5; 95% CI: 1.4–8.8). Additionally, the number of previous hospital stays within the past 6 months raised the probability of a positive CD-PCR by factor 1.6 each (95% CI: 1.1–2.2). This corresponds to previous studies which also determined previous episodes of CDI and treatment with antibiotics as the highest risk factors for a toxigenic CD colonization [34, 35].

There is an established connection between comorbidity and the probability of occurrence and the course of a CDI [5, 17]. Accordingly, we found a small, significant correlation of colonization and the Charlson Comorbidity Index.

Contrary to our expectations, the parameters of the geriatric assessment did not correlate with CD carriage.

Geriatric patients are characterized by a diminished ability to perform activities of daily living (ADL), reduced mobility and strength and often cognitive impairment. Rao et al. have shown that a diminished functional state is connected with the development at least of serious courses of CDI [19]. Therefore, it comes as a surprise to us that neither the Barthel Index, nor the Timed Up and Go Test nor the handgrip strength were associated with a higher probability of CD carriage.

A correlation has been described between dementia and the frequency of MRSA colonization [36]. Thus, a raised probability of CD carriage might have been expected in patients with diminished cognitive abilities, quantified by means of Mini Mental State Examination. Contrary to our expectations, this cannot be confirmed here, either.

Intestinal microbiome is increasingly being recognized as an important health factor. Its potential role for colonization and infection with *Clostridum difficile* seems to be of high importance. The intestinal microbiome is highly influenced by environmental factors. This would explain why in our study factors such as the number and length of hospital stays and the treatment with antibiotics seem to play the key role for colonization with *Clostridium difficile*. The results of the geriatric assessment do not seem to be associated with *C.difficile* colonization. They might be relevant for the outbreak, course and outcome of a symptomatic CDI.

Asymptomatic carriage at admission was a decisive risk factor for the development of a symptomatic infection during the hospital stay. About 87.5% of the patients (7 of 8) who developed a CDI in the course of the study arrived for their hospital stay carrying a toxigenic *C.difficile* strain. In view of this, positive screening for toxigenic *C.difficile* carriage at admission would provide a high predictive value for a later development of a symptomatic CDI, as described in previous studies [25, 26, 31, 37].

Some studies substantiate financial and health benefits to be gained by a *C.difficile* admission screening combined with intensive hygiene interventions/isolation procedures [38, 39]. Consequently, knowing a patient’s carrier status at admission can prove vital and, in positive cases, it would provide various options how to manage the situation: providing efficient hygiene management of these patients would stem the spread of the pathogen, and the number of secondary CDI cases could be reduced. And additional options would arise in the direct medical treatment of these colonized patients with a significantly higher risk for the development of a symptomatic CDI. A therapy with antibiotics and the agents chosen can then be adapted to this risk situation. Should any cases of diarrhoea occur, however, fast counter measures would be possible as this would suggest a high probability of the *C.difficile* toxin as its cause.

A risk adapted screening including high risk patients with previous episodes of CDI, previous hospitalization and previous antibiotic treatment could be reasonable in the light of our results.

There are various methods for testing for *C.difficile* [33]. What is important for efficient admission screening is a fast result. The gold standard of CDI diagnostics – the stool culture – takes several days to show a result. Using PCR from stool samples, as in our study, achieves a timely detection of *C.difficile* carriers. It has already been shown that rectal swabs taken at admission and tested using PCR following a selective broth preamplification can be used for even faster diagnostic [34, 40]. A disadvantage of the PCR screening test, however, is its relatively high price.

One limitation of our study is the fact that we failed to obtain a stool sample in many patients who had already consented to participate (183 of 445). The reason for this was in most cases that the patients or the nurses forgot to take a stool sample. The distribution of Barthel Index and Mini Mental State Examination in both groups was comparable.

A second limitation is that patients in geriatric wards are a heterogeneous population. About 80% were transferred from other departments for further rehabilitation whereas only about 20% were admitted direct. However, this is a real-life scenario.

The third limitation is that we were only able to monitor the patients for the time they spent in hospital. Therefore, conclusions about the development of a CDI have to be drawn with caution. Colonization seemed to be a clear risk factor for the development of a CDI. To what extent the risk factors identified for colonization also play a role as risk factors for infection cannot be evaluated due to limited statistical power.

As a further limitation, the sample size calculation of 250 patients was based on an assumed CD carrier rate at admission of 20% and an in-house CDI rate of 4% for geriatric patients. The CD prevalence at admission in our study reached only 16.4% and the infection rate 3.1% (8 of 262 patients).

Our study is the first to show a high rate of colonization in geriatric in-patients. With CDI rates and recurrences on the increase in this population [41, 42], the usefulness of a PCR screening in a geriatric risk population should be evaluated in a prospective trial.

## Conclusions

This is the first study to show the rate of asymptomatic carriers in a geriatric unit (16.4%), which lies between the previously shown rates of other hospital units and those of care facilities. The parameters of geriatric assessment do not offer any option to predict carriage. This study, too, finds that the highest risk factors are previous episodes of CDI, previous treatment with antibiotics, and previous hospital stays.

Carriage at admission is a huge risk factor for the development of a symptomatic CD infection during the hospital stay. One in every 6 colonized patients (16.3%) falls ill during the stay, or in other words, the majority of patients developing CDI during their stay has arrived colonized for admission (87.5%). This underlines the possible importance of screening for carriage in geriatric units, especially in high-risk patients.

## Abbreviations

95% CI: 95% confidence interval
ADL: Activities of daily living
CD: *Clostridium difficile*
CDI: *Clostridium difficile* infection
CGA: Comprehensive geriatric assessment
EIA: Enzyme immunoassay
GDH: Glutamate dehydrogenase
MMSE: Mini Mental State Examination
PPI: Proton pump inhibitor
